# Umbilical cord milking-benefits and risks

**DOI:** 10.3389/fped.2023.1146057

**Published:** 2023-04-18

**Authors:** Jenny Koo, Hasan Kilicdag, Anup Katheria

**Affiliations:** ^1^ Neonatal Research Institute, Sharp Mary Birch Hospital for Women & Newborns, San Diego, United States; ^2^ Divisions of Neonatology, Baskent University Faculty of Medicine, Ankara, Türkiye

**Keywords:** umbilical cord milking, delayed cord clamping, placental transfusion, infants, newborn

## Abstract

The most common methods for providing additional placental blood to a newborn are delayed cord clamping (DCC) and umbilical cord milking (UCM). However, DCC carries the potential risk of hypothermia due to extended exposure to the cold environment in the operating room or delivery room, as well as a delay in performing resuscitation. As an alternative, umbilical cord milking (UCM) and delayed cord clamping with resuscitation (DCC-R) have been studied, as they allow for immediate resuscitation after birth. Given the relative ease of performing UCM compared to DCC-R, UCM is being strongly considered as a practical option in non-vigorous term and near-term neonates, as well as preterm neonates requiring immediate respiratory support. However, the safety profile of UCM, particularly in premature newborns, remains a concern. This review will highlight the currently known benefits and risks of umbilical cord milking and explore ongoing studies.

## Introduction

An estimated 140 million neonates born worldwide each year are treated with various umbilical cord management techniques (1). The importance of providing additional blood during the birth transition cannot be overstated. During pregnancy, fetal blood volume is approximately 110–115 ml/kg of fetal weight (2). Only 10% of the fetal cardiac output goes to the pulmonary circulation, whereas 30% to 50% is directed toward the placenta for gas exchange (3). After birth, as the lungs expand and the capillary beds around each alveolus are filled with blood, there is a significant increase in cardiac output to the lungs (4, 5). The placenta serves as an ideal blood reserve intended to supply this sudden shift in blood volume. Blood flow through the umbilical cord typically lasts several minutes after delivery which help maintain hemodynamic stability and red cell volume (6). Early observational studies demonstrated that the infant to placenta blood volume ratio increased from 67%/33% at birth to 87%/13% at the end of placental transfusion (7). The amount of placental transfusion that occurs may be influenced by various factors, such as uterine contractions, delivery method, length of time from birth to cord clamp, and possibly gravity and the newborn's spontaneous respirations (8). Placental transfusion is an essential part of the neonatal transition as the increased blood volume affects all organ systems.

Delayed cord clamping (DCC), delayed cord clamping with resuscitation (DCC-R) and umbilical cord milking (UCM) are different approaches to provide placental transfusion after delivery. DCC may be challenging to apply in infants with poor respiratory effort who may require resuscitation or are at risk for hypothermia. Conversely, DCC-R and UCM allow for immediate resuscitation and warming after birth. Compared to DCC-R, UCM poses fewer logistical challenges (equipment, personnel training) and can be more broadly applicable to all neonates worldwide. UCM enhances overall blood volume by 20%–30%, but the limited information on the physiological and hemodynamic impacts of UCM is concerning (4). Gaps in knowledge render UCM difficult to recommend universally. We will discuss the advantages and limitations of UCM in this review.

## Cord management strategies

### Overview of cord management strategies

Cord management plays a vital role in blood volume transfer as well as in hemodynamic stability during transition (7, 9–11). DCC is now widely adapted as the standard of care and is the recommended practice for vigorous neonates by multiple national and international governing bodies (ILCOR (12), WHO (13), SOGC (14), EAPM (15), AAP (16)). DCC can provide up to one-third of a term neonate's total blood volume without increasing the risk of maternal hemorrhage in either vaginal or cesarean section deliveries (17). Animal models have shown negative impacts of early cord clamping (ECC) such as higher pulmonary artery pressure and reduced cardiac output, while demonstrating DCC after lung aeration is established can reduce pulmonary vascular resistance and improve oxygen delivery (18).

In preterm neonates, animal models suggested a potential reduction in the risk of intraventricular hemorrhage since DCC allows for smoother hemodynamic transition, better cardiac output, and less carotid artery pressure fluctuations (19). A randomized control trial of 1,566 preterm neonates randomized to DCC vs. ECC showed a reduction in hospital death following DCC (20). However, multiple meta-analysis did not show differences in the rates of major morbidities including severe IVH, retinopathy of prematurity (ROP), necrotizing enterocolitis (NEC) or late onset sepsis (LOS) (21, 22). Potential barriers to optimizing placental transfusion in preterm neonates is their need for immediate resuscitation and need for respiratory support to establish lung aeration prior to cord clamping (23).

DCC with resuscitation with an intact cord (DCC-R) has been shown to be feasible for providing placental transfusion in preterm neonates in need of respiratory assistance or in non-vigorous term and near-term neonates (24). Studies comparing DCC alone to DCC-R in preterm neonates showed no differences in the rates of severe IVH, NEC, ROP, or hemodynamic markers on echocardiography (25). The benefits of DCC-R in preterm neonates may be limited by inadequate lung recruitment when relying on non-invasive modes of respiratory support during resuscitation with intact cord. In contrast to preterm animal models that are sedated and invasively ventilated, preterm neonates in the delivery room are resuscitated with non-invasive respiratory modalities. Preterm rabbit models showed that resuscitation with non-invasive interfaces such as a face mask is hindered by a larynx and epiglottis that is closed three-quarters of the time during resuscitation (26). Ongoing studies exploring DCC-R vs. DCC in preterm neonates include the VentFirst trial (NCT02742454), the Baby-DUCC trial (ACTRN12617000610336), the ABC3 Trial (NCT03808051). There is a need for additional studies to elucidate the effectiveness of DCC-R for improving immediate and long-term outcomes.

Resuscitation with an intact cord can be done using simple equipment for providing respiratory support while stabilizing temperature, as is the case in developing countries (27). However, a survey has shown that clinicians view DCC-R using a resuscitation trolley as the “same”, “better”, or “much better” than conventional equipment (28). Whether standard resuscitation equipment is used, or if specialized resuscitation trolleys are employed, DCC-R warrants special training as well as the need to consider the sterility of the field in the case of C-section deliveries (29) Resuscitation trolleys with the ability to provide heat and respiratory support are often utilized, and the personnel tasked with the resuscitation must be trained. Multiple trolleys have been developed, with one being FDA-approved and several others actively being tested (30). Due to a number of logistical challenges and requirements, UCM has been explored as an alternative to DCC as a means to provide placental transfusion without delaying resuscitation. Moreover, preterm lamb models showed that UCM with placental refill offered the most transfer of blood volume (median net transfer 8.8 ml/kg) compared to UCM without placental refill or DCC (31). Table 1 summarizes some key features, benefits, and limitations of UCM and DCC.

**Table 1:** Comparing umbilical cord milking to delayed cord clamping.

	Umbilical Cord Milking (UCM)	Delayed Cord Clamping (DCC)
Variations in practice	• Intact-cord UCM (I-UCM) and cut-cord UCM (C-UCM) • Both beneficial over ECC • I-UCM with greater benefits than C-UCM	• DCC is the standard of care for vigorous neonates. • DCC with resuscitation (DCC-R) may reduce any delays to resuscitation
Mode of delivery	• UCM may be the superior cord management strategy in Caesarian sections (C-sections)	• DCC and DCC-R may deliver less placental blood with anesthetics and uterotonics frequently used in C-sections
Maternal outcomes	• No differences in maternal complications • Reduces time with open uterus compared to DCC and DCC-R	• No differences in maternal complications
Hemoglobin and ferritin	• Higher hemoglobin and ferritin than ECC, fewer transfusions needed • Higher markers of blood cell volume at a later time compared to DCC (∼6 weeks)	• Higher hemoglobin and ferritin than ECC, fewer transfusions needed
Hyperbilirubinemia	• No difference in need for phototherapy	• No difference in need for phototherapy
Hemodynamics	• Improve systemic and cerebral perfusion in comparison to ECC • Reduce pulmonary vascular resistance; effects amplified by respirations • greater risk of blood flow swings compared to DCC	• Improve systemic and cerebral perfusion in comparison to ECC • Reduce pulmonary vascular resistance; effects amplified by respirations
Pluripotent cell transfer	• UCM transfers higher levels of CD34+ stem cells compared to DCC	• Less transfer of CD34+ stem cells compared to UCM
Survival and Long term outcomes	• Limited data, likely comparable or superior to DCC in 2-year cognitive and linguistic scores	• Improved survival to discharge compared to ECC • Lower incidence of neurocognitive impairment at 2-year follow up compared to ECC
Limitations	• Potential IVH risk in preterm neonates <28 weeks’ gestational age	• DCC may delay resuscitation • DCC-R may require special equipment or training

### Cord management strategies during different modes of delivery and maternal outcomes

The mode of delivery may impact the efficacy of placental transfusion. A meta-analysis suggests that anesthesia and surgical procedures interfered with the uterine muscles' ability to contract actively and thereby limiting placental transfusion at birth. Term neonates born by cesarean section (C-section) received less placental transfusion compared to infants born by vaginal delivery, and thereby have lower hematocrit, hemoglobin, and erythrocyte levels (32).

Several randomized controlled trials have demonstrated preterm infants delivered by C-section undergoing UCM vs. DCC had better systemic blood flow, blood pressure, hemoglobin levels, and urine output in the first 72 h of life (33–35). UCM has been found to be feasible in term infants born by C-section and did not result in higher incidence of phototherapy, symptomatic polycythemia, NICU hospitalizations, or readmissions for phototherapy (36). UCM may be a good alternative in elective C-section to optimize placental transfusion (37).

Maternal outcomes do not differ with UCM vs. DCC (38, 39). Primary post-partum hemorrhage (PPH) was present in 3.1% of women in the DCC arm and 2.3% of mothers in the UCM arm. (*p* = 0.719) (38). UCM results in a shorter time with an open uterus at cesarean section and thereby unlikely to increase maternal adverse events.

### Approaches to umbilical cord milking: intact vs. cut cord milking

**Figure 1:**
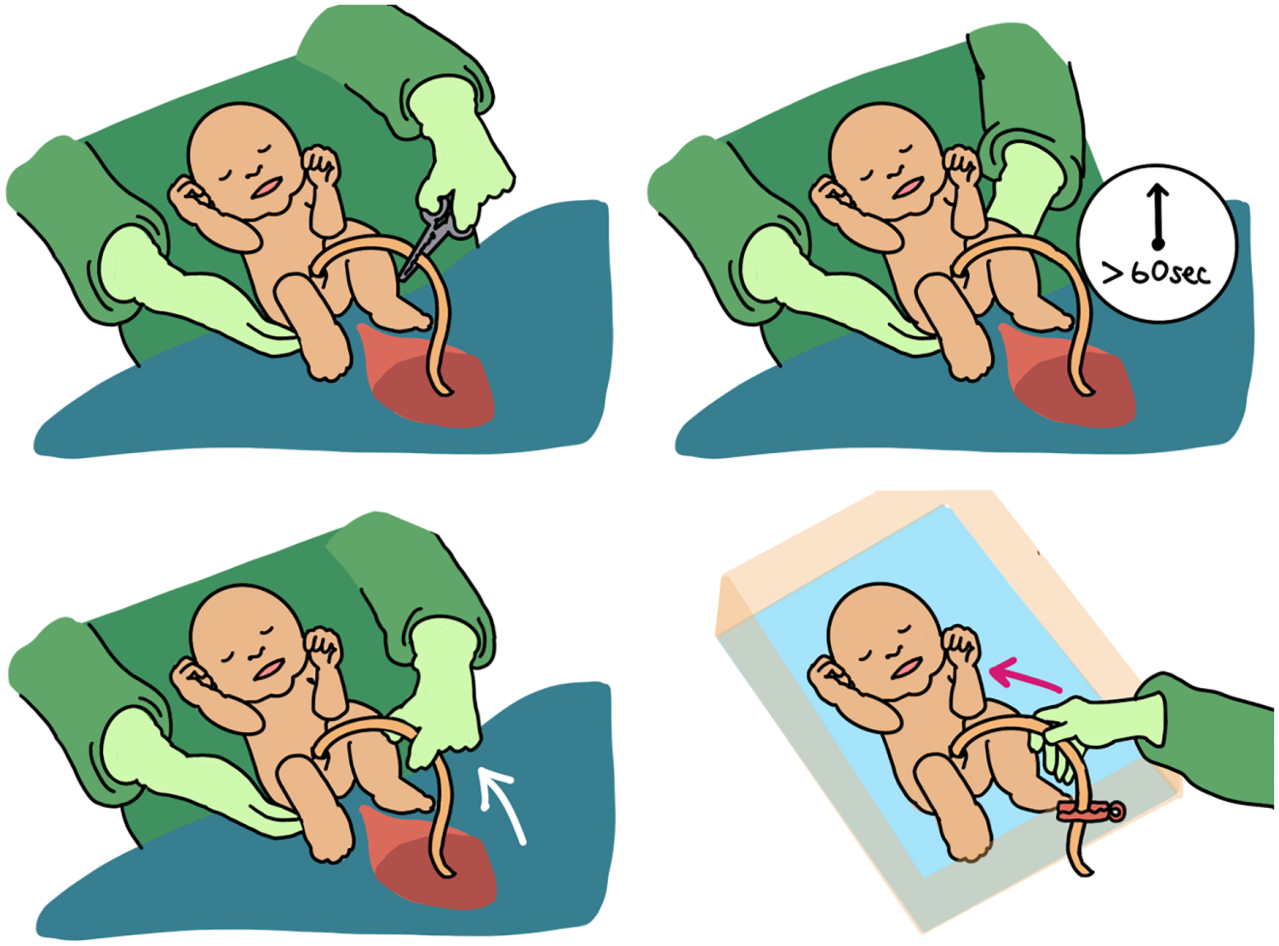
Early cord clamping (ECC) vs delayed cord clamping (DCC) vs. intact cord umbilical cord milking (I-UCM) vs. cut-cord umbilical cord milking (C-UCM). Different methods of cord management are depicted here.

There is presently no agreement on the ideal method for cord milking (Figure 1). Intact umbilical cord milking is performed by manually expressing umbilical cord blood three to four times at a rate of 10 cm per second down a 20–30 cm section of the umbilical cord (40). Cut cord milking (C-UCM) is performed by clamping the cord 10–30 cm away from the infant so that the infant is moved to a resuscitation bed before the cut-cord is milked (41). The clamped umbilical cord is milked once towards the infant while receiving respiratory support. Both cut and intact cords have been used for umbilical cord milking with varied results (42, 43).

Until recently it has not been clear whether milking the cut vs. intact cord provides a different amount of transfusion (40). McAdams et al. demonstrated that milking the intact cord (I-UCM) in term infants provided a greater volume of blood compared to milking a cut cord (40). Moreover, longer cord lengths provided greater blood volumes transferred to the neonate. C-UCM did not reduce newborn morbidities or enhance hemoglobin levels in a sample of 106 preterm infants born at 35 weeks of gestation when compared to historical controls who underwent ECC (41). A large randomized controlled trial demonstrated I-UCM resulted in higher hemoglobin and hematocrit levels at 48 h of age compared with the ECC group (44).

A systematic review and meta-analysis included nine RCTs (*n* = 1,632) that compared I-UCM, C-UCM and DCC in term and late-preterm babies. C-UCM increased hemoglobin levels in term and late-preterm babies at 30 min when compared to DCC. Both UCM methods improved hemoglobin levels at 48–72 h when compared to DCC. Only I-UCM was associated with higher hemoglobin at a later time point (6 to 8 weeks postnatally) when compared to DCC. There was no variation in serum ferritin levels (45). Another study found that C-UCM was safe in term and near-term newborns and produced greater hemoglobin levels at 6 weeks of life than ECC (46). To date, literature suggests superiority of I-UCM over C-UCM, but any method of UCM has clinical benefits over ECC. For that reason, and given the safety profile of C-UCM, it is an acceptable variation of UCM in term and near-term newborns.

## Benefits of umbilical cord milking

### Transfusion, anemia, polycythemia, and jaundice

Children with iron deficiency have delayed cognitive development, behavioral problems, and decreased motor development (47–49). Iron deficiency may be decreased by cord management and placental transfusion at delivery. The outcomes in premature newborns receiving UCM are mixed; some report increased ferritin and hemoglobin levels, while other studies report no change (50–55). The need for blood transfusion was significantly lower in the I-UCM group when compared to ECC in a systematic review, with a number needed to treat (NNT) of four to prevent one packed red blood cell (pRBC) transfusion (56). Another systematic review of six trials (*n* = 587 preterm infants) also reported fewer pRBC transfusions with UCM compared to ECC (57). Lastly, a systematic review from three studies that compared UCM and DCC in 650 term babies delivered vaginally or by cesarean section had similar hemoglobin and ferritin levels in the short term period. However, UCM was found to have a higher hemoglobin level at six weeks of age (58).

There are conflicting data on the association between UCM and polycythemia and hyperbilirubinemia. While some small studies suggest higher rates or longer duration of phototherapy, many contemporary observational and randomized control trials have refuted these concerns (50, 59, 60). The most recent Cochrane analysis found no difference for the need for phototherapy with DCC or UCM (8 studies, 495 infants) (21). A systematic review of UCM and DCC in preterm infants found two studies with blood bilirubin levels at 48 h of life were reported in 385 newborns and found no difference (58). Another RCT of 280 infants between 28 and 37 weeks found a higher incidence of neonatal jaundice with DCC (15.6%) compared to the UCM (6.8%) (38).

In a 3-arm randomized controlled trial of 300 term babies, Yadav et al. compared the effects of DCC with C-UCM or I-UCM. The number of patients receiving phototherapy for jaundice and the serum bilirubin level at 48 h were comparable among all three groups (61). A cluster crossover trial comparing early cord clamping to cord milking in non-vigorous infants (*N* = 1,730) demonstrated a higher serum bilirubin with cord milking but no increase in the need for phototherapy (62). Bilirubin is a the most abundant antioxidant in the newborn, and may be associated with improved neurodevelopmental outcomes in infants with hypoxic ischemic encephalopathy, so higher levels without increased need for therapy may even be neuroprotective (63). No large studies or systematic reviews to date have demonstrated an increase in the need for phototherapy treatment or exchange transfusion with umbilical cord milking compared to delayed or early cord clamping.

### Hemodynamic parameters, cerebral oxygenation, and breathing

UCM and DCC enhance systemic and cerebral perfusion in comparison to ECC, both of which may be neuroprotective. Both UCM and DCC have shown some modest promise in increasing brain oxygenation and hemodynamic parameters on echocardiography (60, 64–67).

**Figure 2:**
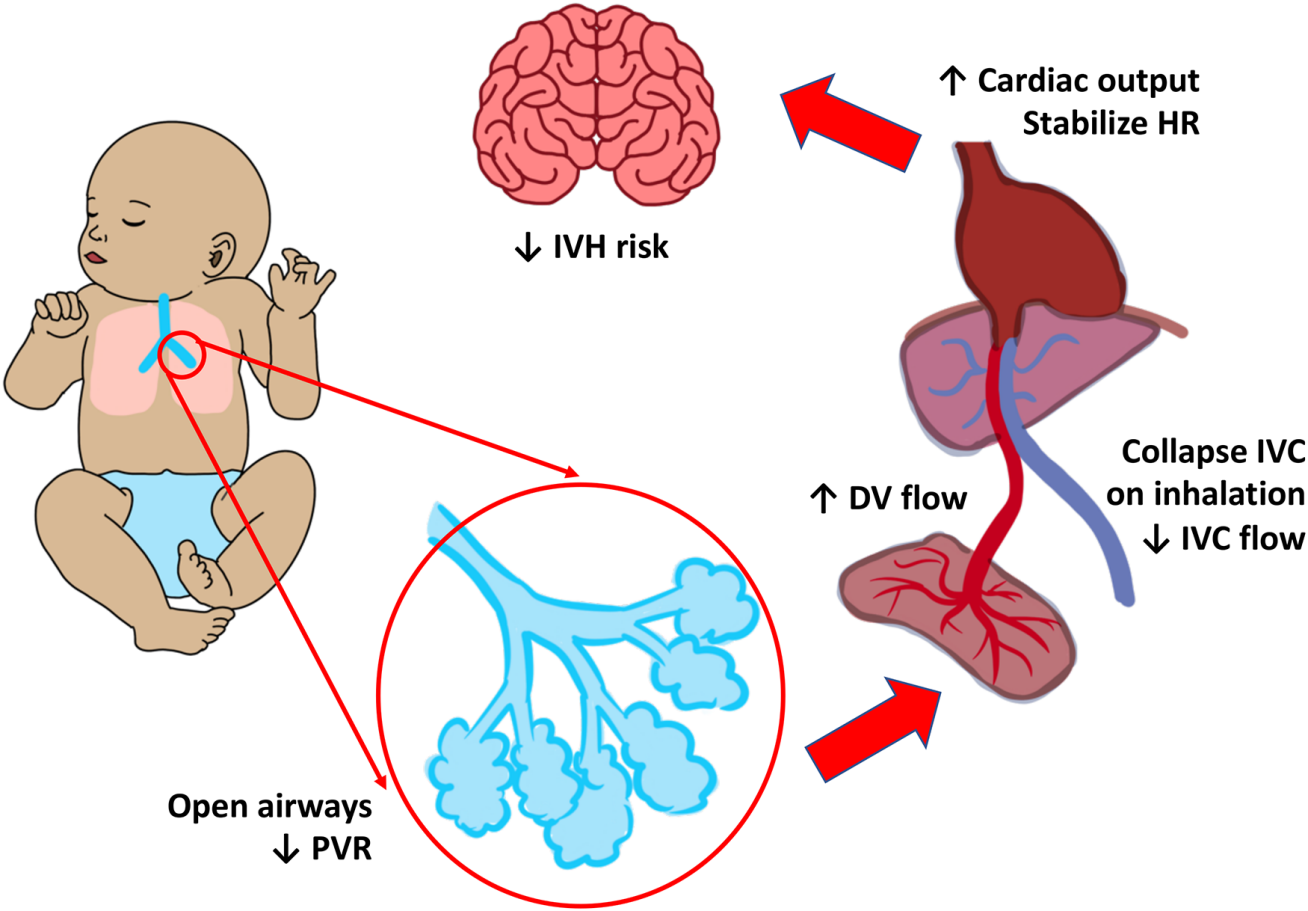
Cardiorespiratory changes during respiration before cord clamping. Respiration and lung recruitment drops the pulmonary vascular resistance (PVR), collapses the inferior vena cava (IVC), and thereby increases the flow of blood from the ductus venosus (DV). This increases cardiac output (CO) which in turn stabilizes the heart rate (HR), blood pressure (BP), and reduce the risk of intraventricular hemorrhage (IVH).

Cord management and spontaneous or assisted ventilation concertedly impact the degree of placental transfusion to the newborn (68). It is now understood that the onset of spontaneous respirations and the opening of the pulmonary vascular bed are key factors in placental blood transport. In a small observational study, spontaneous breathing was associated with collapse of the inferior vena cava (IVC) and increased anterograde flow in the ductus venosus and hepatic vein during inspiration. When breathing begins, lung expansion increases pulmonary blood flow, which makes pulmonary venous return the main source of preload for the left ventricle in place of umbilical venous return. The heart rate stabilizes when cardiac output rapidly rises (Figure 2) (69). In contrast, ECC lowers cardiac output in newborns who are apneic and hypoxic. Limiting the rise in cardiac output exposes the newborn to the combination of ischemia and hypoxia since higher cardiac output mitigates the consequences of hypoxemia (70).

As noted above, animal models suggest that adequate ventilation is protective against abnormal fluctuations in lambs receiving UCM (71). However, it is possible that UCM may stimulate spontaneous breathing in human neonates as contrasted to anesthetized lamb models. Preterm newborns receiving ECC at delivery had lower heart rates and oxygen saturation than preterm infants getting UCM, and required more oxygen and ventilation in the first five minutes of life suggesting the UCM may play a role in respiratory adaptation (72).

In term infants who were randomly assigned to receive C-UCM or DCC, Jaiswal et al. evaluated the blood flow velocity and Doppler indices of the middle cerebral artery (MCA) between 24 and 48 h after birth, as well as hemodynamic parameters (such as blood pressure, heart rate, and respiratory rate) at 30 min, 24 h, and 48 h of life. They noted that both the C-UCM and the DCC groups had similar cerebral blood velocities and cranial Doppler indices. Similarly, there was no discernible difference between the two groups in terms of the MCA cerebral blood flow velocity, pulsatility index, and mean resistive index (73).

The higher systemic pressure combined with high pulmonary vascular resistance in non-breathing infants may prevent a rise in pulmonary blood flow during milking. Without a pulmonary pop-off, the rise in aortic pressure may result in fluctuations in cerebral blood flow (8). Understanding how UCM influences hemodynamic parameters is essential. UCM's impact on hemodynamic parameters must be further investigated in different gestational ages and in breathing and non-breathing infants.

### Stem cell transfer

**Figure 3:**
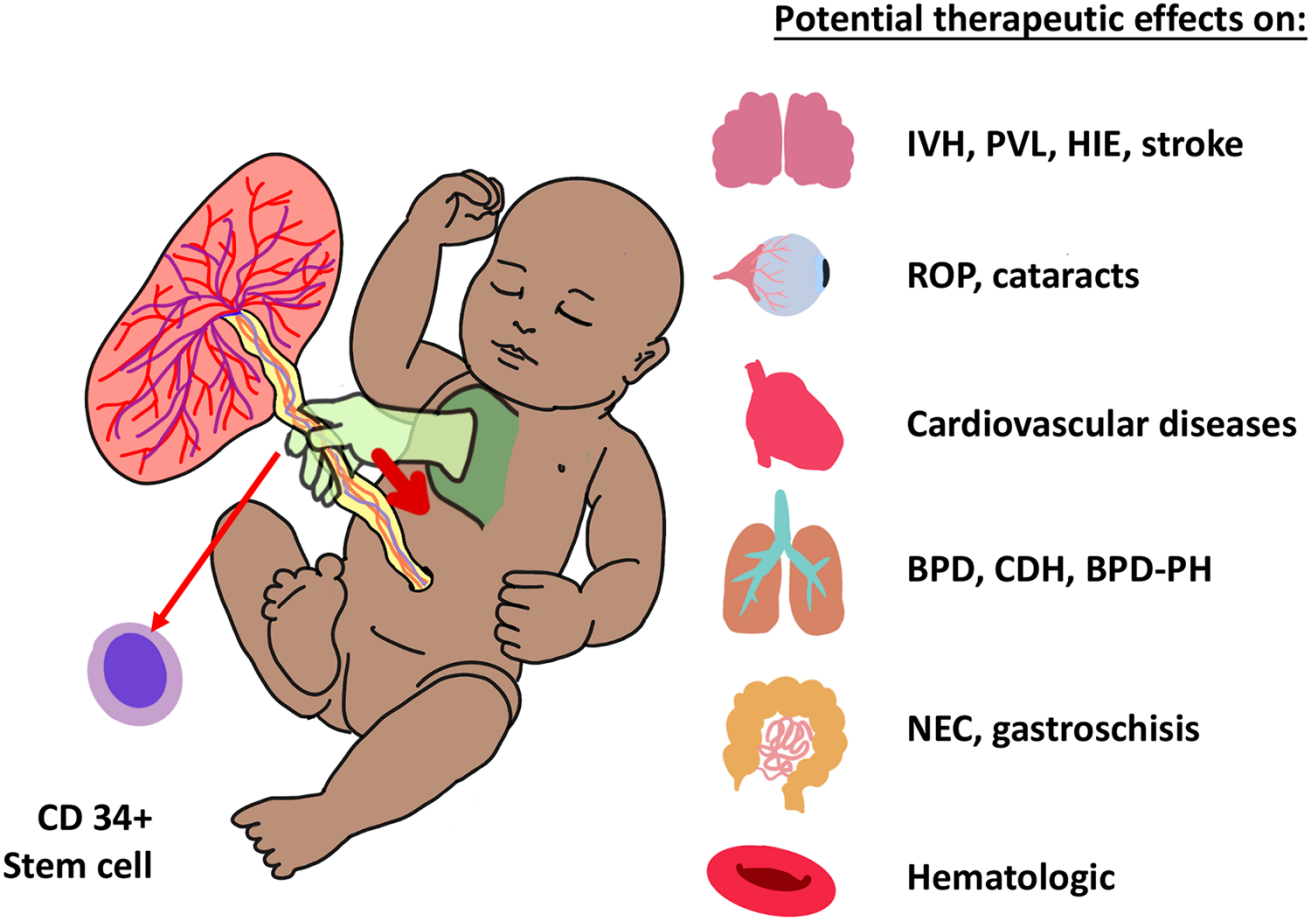
Umbilical cord milking (UCM) increases CD34 + pluripotent stem cells in babies, which have potential benefits as summarized in the figure. IVH, intraventricular hemorrhage; PVL, periventricular leukomalacia; HIE, hypoxic ischemic encephalopathy. ROP, retinopathy of prematurity; BPD, bronchopulmonary dysplasia; CDH, congenital diaphragmatic hernia; PH, pulmonary hypertension; NEC, necrotizing enterocolitis.

Reports from over 80 years ago have suggested that although DCC and UCM lead to increased hematocrit, umbilical cord milking is associated with a sustained increase in red blood cell count compared to DCC. A bedside to bench model studied umbilical cord blood contents in term infants randomly assigned to receive UCM or DCC. UCM blood exhibited higher levels of hematopoietic stem cells (CD34) and improved survival in irradiated mice when compared to DCC blood (74). In an observational study, preterm infants with placental insufficiency had higher CD34 counts following UCM as compared to historical controls who underwent ECC (75). Although not yet proven, stem cell transfusion during UCM has promising applications towards preterm and term infants with organ injury or hematopoietic cell line deficiencies (Figure 3) (76).

### Long-term outcomes

Data on the long-term results of UCM in infants is scarce. Studies to date have described a positive association between neurodevelopmental outcomes and adequate iron storage (77). Katheria et al. demonstrated higher language and cognitive scores at two years of age in patients randomized to UCM compared to DCC, but no difference in rates of neurodevelopmental impairment (78). Rabe et al. observed that preterm children who received UCM at birth had higher or comparable cognitive and linguistic scores to those who received DCC (79). One study compared 56 neonates born between 24 and 27 weeks' gestation and found no differences in neurodevelopmental outcomes between the UCM and ECC groups (54). Larger studies evaluating long term outcomes are needed.

### Cord milking for non-vigorous babies

An important stage in newborn stabilization may be optimum cord management to improve placental transfusion (80). Randomized controlled trials have excluded these infants due to the need for rapid resuscitation. While the difficulties of a high-pressure environment accompany the delivery of a depressed newborn, providing these newborns with an appropriate placental transfusion may be the first and most crucial step in assuring the best prognosis (81). Infants who are at risk of hypoxia may be at risk of not getting this transfusion right away. Asphyxiated infants lose 30% of their final blood volume and 60% of their RBC volume due to ECC (82). The use of ECC in these vulnerable newborns may aggravate hypoxia-ischemia and have negative short- and long-term consequences (83).

In the past, UCM has been used instead of DCC in neonates who were asphyxiated, required urgent resuscitation, or were delivered through C-section (84). UCM makes it possible for a neonate to get a faster placental transfusion, which makes it easier to move the baby from the mother to a warmer bed for more life-saving measures (85). Methods of placental transfusion may aid in the cardiovascular transition and be neuroprotective in newborns who are not in good health. This extra blood improves blood volume and boosts cardiac output, stabilizing pulmonary and cerebral circulation, increasing cardiac preload before the placenta is removed from the circulation, perhaps reducing further ischemia in a newborn already suffering from poor health (81, 86).

However, there may be risks of provide rapid volume in asphyxiated infants. Concerns about volume overload in newborns who have had hypoxic-ischemic events during delivery and increased endothelial activation products entering the infant's circulation with milking of the umbilical arteries have been raised (87).

There have been few studies on UCM in infants with depression. Ram Mohan et al. performed a pilot RCT of 60 non-vigorous preterm infants to either C-UCM or ECC and demonstrated similar clinical outcomes, but infants randomized to C-UCM infants had higher hemoglobin and ferritin levels at six weeks of age (88). Girish et al. performed a quasi-randomized trial of 100 near term and term non-vigorous newborns to either UCM or ECC and demonstrated similar outcomes of HIE death and need for resuscitation (89). However, there is inadequate research to support DCC or umbilical cord milking (UCM) in cases of prenatal distress (80). In a cluster-randomized crossover study (MINVI trial) proposed that UCM, as opposed to early cord clamping (ECC), would decrease admission to the neonatal intensive care unit (NICU) in non-vigorous newborns delivered between 35 and 42 weeks of gestation. While UCM did not result in a decrease in NICU admissions when compared to ECC, it did result in fewer occurrences of moderate to severe hypoxic-ischemic encephalopathy, less therapeutic hypothermia usage, and higher hemoglobin levels. When compared to ECC, there was no evidence that UCM caused any harm. These findings may represent the first randomized controlled trial proof that UCM is possible, safe, and preferable to ECC in non-vigorous newborns (62). An ongoing trial in India aimed to reduce HIE may help determine whether UCM can be efficacious in non-vigorous newborns (NCT03657394).

## Risks with umbilical cord milking

UCM can provide placental transfusion without significantly delaying resuscitation. Despite clinical trials demonstrating its advantages for the short-term outcomes of preterm infants, UCM has not been adopted as an alternative to DCC. There remain concerns about rapid changes in blood flow in extremely preterm infants that may increase the risk for intraventricular hemorrhage (64, 90, 91).

Anesthetized preterm lamb models delivered by C-section have demonstrated oscillations in the systemic arterial pressure and cerebral blood flow with UCM (12, 31, 92). However, asphyxiated preterm lamb models receiving UCM concurrently with ventilation exhibited a decrease in fluctuations of carotid and pulmonary blood flow as compared to UCM alone (71).

The safety of providing UCM to extremely premature infants remain unclear. To date, there is only one randomized controlled trial that has demonstrated an association with UCM and severe IVH compared to delayed cord clamping (93). However, the trial enrolled substantial numbers (*N* = 182) of infants 23–27 weeks' gestation and demonstrated an increase in severe IVH at each of the 10 centers that participated. Whether prior animal data is translatable in these infants is unclear, however there is biological plausibility that the additional blood volume provided by UCM may not be as well tolerated in the extremely preterm infant who lack cerebral autoregulation and have a very fragile germinal matrix which may lead to hemorrhage (8). Unfortunately, most of the literature on preterm infants comes from small studies or mostly mature infants (34, 90, 94, 95). Recent meta-analyses of these studies may also only provide false assurance of safety (56). Retrospective studies have additional problems of bias since sicker infants that receive UCM may go on to develop IVH and die irrespective of their cord management. A 3-arm study comparing cut cord milking, intact cord milking and delayed cord clamping in China may help support or refute current concerns regarding UCM in preterm infants (96).

## Research directions

There is an urgent need for larger randomized controlled trials of cord management powered to detect signals of safety and harm. Studies need to include multiples and assess the safety of delayed cord clamping or milking on neonatal outcomes. Monochorionic placentation also needs to be included if there is no risk for twin-to-twin transfusion. Moreover, variations of the milking practice, including how many times, over how long, allowing for placental refill or not, and whether cut cord milking provides similar or greater benefit than milking the intact cord. The impact of UCM on different populations needs to be further delineated, including neonates of different gestational ages, vigorous neonates with spontaneous respirations, and depressed, non-breathing neonates requiring positive pressure ventilation.

Translational studies are paramount for elucidating the physiologic changes that take place during umbilical cord milking in order to identify additional risks and benefits of this practice.

The study and application of cord milking in low-middle income countries where the incidence of HIE is 10-fold higher is urgently needed.

## Conclusions

Understanding the benefits and risks of UCM can be challenging, even when considering systematic reviews and meta-analyses. UCM may be safe for many infants, but exercise caution in subgroups such as the extremely preterm infant. Cord milking may be of particular benefit in conditions where DCC is contraindicated such as abruptions or maternal instability. Future studies will hopefully provide additional guidance as to which populations may benefit from UCM.
